# Supplementary material for: Homopolymer self-assembly of poly(propylene sulfone) hydrogels via dynamic noncovalent sulfone–sulfone bonding
Source: Nat Commun. 2020 Sep 29;11:4896. doi: 10.1038/s41467-020-18657-5 (PMC7525563; doi:10.1038/s41467-020-18657-5)
Supplement: Supplementary file 1 — Supplementary Information [file 41467_2020_18657_MOESM1_ESM.pdf]

Supplementary Information

Homopolymer self-assembly of poly(propylene sulfone) hydrogels via dynamic noncovalent sulfone-sulfone bonding

Fanfan Du, Baofu Qiao, Trung Dac Nguyen, Michael Vincent, Sharan Bobbala, Sijia Yi, Chamille Lescott, Vinayak P. Dravid, Monica Olvera de la Cruz, Evan Alexander Scott*

*Correspondence to: Email: evan.scott@northwestern.edu (E.A.S.)

Supplementary Methods

Chemical reagents. All chemical reagents were purchased from Sigma-Aldrich St. Louis, MO, USA, unless stated otherwise. Recombinant A. victoria GFP protein (from the Jewett Lab), FITC-RNA and FITC-DNA conjugates (from the Mirkin Lab) were obtained as generous gifts from other Labs at Northwestern University.

Polymer synthesis. PPSU was synthesized from complete oxidation of the corresponding PPS. Mixing PPS with 30% of hydrogen peroxide (1 g PPS per 100 mL of H_2O_2 solution) and shaking the mixtures led to a homogeneous solution overnight. Lyophilization of the obtained solution resulted in white shiny solids of PPSU without requirement for further purification. It is worthwhile to note that an oxidation intermediate (sulfone/sulfoxide mixtures) can be collected by

precipitating the homogeneous solution in THF. Apparent differences were discovered for $PPSU_{20}$ and the random copolymers of sulfoxides and sulfones. First, $PPSU_{20}$ powders are demonstrated by WAXD to be crystalline while the sulfoxides/sulfone mixtures are mostly amorphous. Second, the sulfoxides/sulfone mixtures are readily dissolved by water while $PPSU_{20}$ solids are completely insoluble in water.

All-atom explicit solvent molecular dynamics simulations. Classical all-atom molecular dynamics simulations were performed using the CHARMM 36m force field[1]. The recommended CHARMM TIP3P water model[2] was applied with the structures constrained using the SETTLE algorithm[3]. The simulations were performed using the package GROMACS (version 2016.3)[4]. In all the simulations, the degree of polymerization (DP) of 20 was employed for the polymer chains, the same as that $PPSU_{20}$ in the experiments. The polymer chains were created in the extended form. Six extended $PPSU_{20}$ chains were randomly dissolved in each of the three water boxes initially. Whereas in the DMSO systems, the initially extended $PPSU_{20}$ chains were equilibrated in a vacuum, forming coiled configurations, and were then dissolved in the DMSO boxes. The initial configurations are provided in Supplementary Figure 3. See Supplementary Table 2 for the components of the systems. For both solvent conditions, three parallel simulations were performed.

The system potential energy was first minimized using the steepest descent algorithm, followed by the equilibration of 1 ps in the NVT ensemble (constant number of particles, volume and temperature). Subsequently the NPT ensemble (constant number of particles, pressure and temperature) was applied. An equilibration of 10 ps using the time step of 1 fs was followed by another equilibration of 0.1 ns using a time step of 2 fs in the DMSO system, or 2.5 fs in the aqueous system. Subsequently long equilibration simulations were performed. The periodic boundary conditions were applied in all three dimensions. The neighbor searching was performed

up to a cut-off distance of 1.2 nm by means of the Verlet particle-based approach and was updated every 20 time steps. The potential-switch method was applied for the short-range Lennard-Jones (LJ) 12-6 interactions from 1 nm to 1.2 nm. The short-range electrostatic interactions were calculated up to 1.2 nm, and the long-range electrostatic interactions were calculated by means of the Particle Mesh Ewald algorithm[5]. A time step of 2 fs (2.5 fs) was employed by constraining all the covalent bonds using the LINCS algorithm[6] in the DMSO system (water system). The temperatures of the $PPSU_{20}$ solute and the solvent molecules were separately coupled using the Nosé-Hover algorithm (reference temperature 298 K, characteristic time 1 ps). The isotropic Parrinello-Rahman barostat was utilized with the reference pressure of 1 bar, the characteristic time 4 ps and the compressibility 4.5×10^{-5} bar^{-1}. Each of the simulations run 200 ns, with the last 50 ns employed for the data collection and analysis. The final simulation snapshots are presented in Supplementary Figure 3. The convergence of the simulations was justified by the calculations of the potential energies and the sizes of the polymer chains, both as a function of the simulation time. The polymer structures were calculated using the end-to-end distance and the persistence length.

Additionally, control simulations were performed. In the control simulations, initially extended polymer chains were employed for both DMSO and water solvent systems. The annealing simulations were performed to speed up the convergence of the equilibrations[7]. In the annealing simulations, the temperatures of polymer and solvent (DMSO or water) were separately coupled. The temperatures started at 298 K initially, which increased to 353 K within 1 ns. They stayed at 353 K for 9 ns, then dropped to 298 K within 1 ns. Finally, the temperatures stayed at 298 K for another 9 ns. Therefore, each annealing cycle lasted 20 ns. 5 annealing cycles were performed for the DMSO systems (100 ns in total), and 8 cycles (160 ns in total) for the water systems.

Subsequently, the production simulations were performed for 40 ns each. Agreements were found in regards with the polymer chain distribution (molecularly dissolved in DMSO and aggregated in water), the polymer structures (end-to-end distance and persistence length) and the radial distribution function of the polymer sulfur atoms.

Calculation of the dipolar energy between neighbor charge-neutral units from atomistic molecular dynamics simulations. In all the atomistic simulations, the PPSU repeat units and the solvent (DMSO or water) molecules are charge-neutral. The monopole interactions between them could be reasonably expected negligible. By following a previous work[8], we calculated the dipolar interactions between the neighbor units. Each PPSU repeat unit is defined as one charge-neutral unit, as well as one DMSO molecule and one H_2O molecule. The dipolar interaction energy between the charge-neutral units is calculated as below:

1) The sulfur atoms of PPSU repeat units, the sulfur atoms of DMSO and the oxygen atoms of water molecules are employed as the center of the corresponding units. The radial distribution functions between these atoms are calculated (Supplementary Figure 8). Note that for the S(PPSU)-S(PPSU) calculations all the intramolecular correlations within 5 consecutive repeat units were excluded based on the calculated persistence of 4.4 repeating units in water (Supplementary Table 3). The first minima were chosen to define the upper distance of the neighbors, which was 6.7 Å for S(PPSU)-S(PPSU), 7.1 Å S(PPSU)-S(DMSO) and 5.3 Å S(PPSU)-O(H_2O).
2) The dipole moment of PPSU repeat units, DMSO molecules and water molecule were calculated via

$$\vec{\mu} = \sum_i [(\vec{x_i} - \overrightarrow{x_{center}}) \cdot q_i] \quad \text{(Supplementary Equation 1)}$$

$\overrightarrow{x_i}$ denotes the coordinate of atom i with the atomic partial charge q_i. $\overrightarrow{x_{center}}$ stands for the coordinate of the center atom of S(PPSU), S(DMSO) or O(H_2O). The average dipole moments were calculated to be 2.347 D for the CHARMM TIP3P water model, the same as the reported value of 2.347 D[9]. The dipole moment of DMSO was calculated to be 5.22 D, in consistent with the reported value of 5.11 D in the original literature where the DMSO CHARMM force field were originally presented, and around 20% larger than the experimental value[10]. The dipole moment was calculated to be 6.534 D for PPSU repeat units.

3) The dipolar interaction energy between units i and j could be thus obtained by

$$U = \frac{1}{4\pi\varepsilon_0 r^3}\left[\overrightarrow{\mu_i} * \overrightarrow{\mu_j} - 3(\overrightarrow{\mu_i} * \hat{r})(\overrightarrow{\mu_j} * \hat{r})\right] \qquad \text{(Supplementary Equation 2)}$$

where $\overrightarrow{\mu_i}$, $\overrightarrow{\mu_j}$ is the dipole moment of units i and j, respectively; $\hat{r}$ stands for the unit vector between the centers of the two units. Here the units refer to PPSU repeat units, DMSO molecules and water molecules. The cutoff distances were defined in step 1.

4) The steps 2 to 3 were performed for all the dipole-dipole interactions of PPSU-PPSU and PPSU-DMSO in the DMSO solution, and PPSU-PPSU and PPSU-water in the aqueous solution. The distribution of the calculated dipolar energies is provided in Supplementary Figure 6.

Humidity induced-aggregation of $PPSU_{20}$ in DMSO. DMSO solutions of $PPSU_{20}$ were exposed to humidity in air and the phase transition was tracked. Sol-to-gel phase transition was observed for a highly concentrated solution (200 mg mL^{-1}) overnight and a low concentration solution (25 mg mL^{-1}) became cloudy in 3 days. As the cloudy solution was allowed to age further (110 days), fluffy precipitates (can be observed in 7 days) were obtained by centrifugation. The fluffy precipitates were demonstrated by WAXD to be mostly amorphous (Supplementary Figure 9).

After dispersing in water, these fluffy precipitates were recollected by centrifugation. In samples treated this way, WAXD showed an increased crystallinity in Supplementary Figure 9.

CryoTEM imaging. Samples were prepared by applying 3 μL of sample (5 mg mL^{-1}) on pretreated holey or lacey carbon 400 mesh TEM copper grids (Electron Microscopy Sciences). Following a 3 s blot, samples were plunge-frozen (Gatan Cryoplunge 3 freezer). Images of samples entrapped in vitreous ice were acquired using a field emission transmission electron microscope (JEOL 3200FS) operating at 300 keV with magnification ranging from 2,000 × to 12,000 × nominal magnification. Digital Micrograph software (Gatan) was used to align the individual frames of each micrograph to compensate for stage and beam-induced drift. Any further image processing conducted on the aligned frames was completed in ImageJ.

SAXS measurements. SAXS measurements were performed at the DuPont-Northwestern-Dow Collaborative Access Team (DND-CAT) beamline at Argonne National Laboratory's Advanced Photon Source (Argonne, IL, USA) with 10 keV (wavelength λ = 1.24 Å) collimated X-rays. All the samples (5 mg mL^{-1}) were analyzed in the q-range (0.001–0.5 $Å^{-1}$), with a sample-to-detector distance of approximately 7.5 m and an exposure time of 1 s. The diffraction patterns of silver behenate were utilized to calibrate the q-range. The momentum transfer vector q is defined as $q = 4\pi \sin\theta \lambda^{-1}$, where θ is the scattering angle. Data reduction, consisting of the removal of solvent/buffer scattering from the acquired sample scattering, was completed using PRIMUS 2.8.2 software while model fitting was completed using SasView 4.0.1 software package. The core-shell cylinder, vesicle and core-shell sphere models were utilized to analyze the data.

Transmission electron microscopy of negatively stained $PPSU_{20}$ nanostructures. 1.5% uranyl formate (UF) was prepared in ultrapure water. The pH was adjusted to 4.5 by addition of 10 N KOH. 4 μL of nanostructures were applied to glow discharged (25 W, 10 s) formvar carbon film

copper grids (400 mesh, Electron Microscopy Sciences, Inc.). Samples were gently washed twice via passage through ultrapure water, and were negatively stained by passage through two 30 μL volumes of 1.5% UF. Excess stained was removed by blotting each sample with Whatman filter paper. After this procedure, ~0.5 μL stain remains on the grid with an activity of 2.55 × 10^{-5} μCi $grid^{-1}$. Images were acquired at 30,000 × on a JOEL 1400 Transmission Electron Microscope operating at 120 kV.

Energy dispersive X-ray spectroscopy. 1 μL of $PPSU_{20}$ nanobundles (5 mg mL^{-1} in water) were mixed thoroughly with 1 μL of 2% methyl cellulose and 3 μL of water. 5 μL of the mixture was deposited on formvar carbon film copper grids (200 mesh, Electron Microscopy Sciences, Inc.) that were glow discharged to create a hydrophilic surface. After resting for 15 s, the grid was incubated in 1.5% UF for 15 s. Excess stained was wicked away with filter paper. Scanning transmission electron microscopy images were taken with high angle annular dark field (HAADF) mode where bright contrast is indicative of a species with a higher atomic number. In this case, uranium from UF provides the contrast, and the absence of that contrast shows the structure of the embedded sample. EDS maps were taken on a Hitachi HD-2300A dedicated STEM equipped with dual EDS detectors and Thermo Fischer Scientific NSS software. Operating at 200 kV with a 58 μA emission current and an aperture with 75 μm diameter, 90 frames of a 512 × 384 pixel EDS map were collected in EDX Mode. The overall frame time was 10 s with a pixel dwell time of 50 μs. Data was processed by binning with a 17 × 17 kernel size.

Dynamic light scattering. Size distribution and zeta potential of $PPSU_{20}$ nanostructures (1 mg mL^{-1}) were analyzed by Zetasizer Nano (Malvern Instruments) with a 4 mW He-Ne 633 nm laser at 22 °C. The polydispersity index (PDI) was calculated using a two-parameter fit to the DLS correlation data.

Adsorption of FITC-BSA by preformed $PPSU_{20}$ vesicular nanogels. $PPSU_{20}$ vesicular nanogels were prepared by mixing 100 μL of water or aqueous FITC-BSA (1.0 mg mL^{-1}) solution with 100 μL of $PPSU_{20}$ solution (25 mg mL^{-1} in DMSO) under vortex, followed by the addition of 1 mL of water. Nanogels were collected by centrifugation and purified by 2 rounds of resuspension and centrifugation in 1 mL of water. The preformed blank vesicular nanogels were further incubated with 100 μL of aqueous FITC-BSA (1.0 mg mL^{-1}) solution overnight, giving FITC-BSA-adsorbed nanogels after removal of free FITC-BSA by three rounds of centrifugation and resuspension in water. Blank nanogels, FITC-BSA-loaded nanogels, and FITC-BSA-adsorbed nanogels were resuspended in 1 mL of water for fluorescence measurements or as the stocking solutions for quantitative fluorescence analysis in sodium hydroxide solution (0.2 N).

Cell culture. RAW 264.7 cells (murine macrophage cell line) were acquired from American Type Culture Collection (ATCC, Rockville, MD, USA) and used for cell culture experiments. This cell line was cultured in Dulbecco's Modified Eagle's Medium (DMEM) supplemented with 10% fetal bovine serum (FBS), penicillin (100 IU mL^{-1}) and streptomycin (100 μg mL^{-1}) at 37 °C with 5% CO_2.

Cell viability. Tetrazolium (MTT) assay and flow cytometric assessment were performed to investigate cell viability upon nanostructures treatment. In MTT assay, RAW 264.7 cells (3×10^5 cells mL^{-1}, 100 μL) were plated in each well of a 96-well plate and left overnight in the incubator for adherence. The adhered cells were treated with different concentrations of $PPSU_{20}$ nanogels (1, 0.5, 0.25 and 0.125 mg mL^{-1}). After incubation for 24 h, all wells were added with MTT (5 mg mL^{-1} in PBS, 10 μL) and further incubated for 4 h. The resultant formazan crystal deposition in each well was dissolved in DMSO (200 μL) and the absorbance was measured at 560 nm. All the samples were analyzed in quadruplicates. The percentage cell viability was calculated as: % cell

viability = (OD of treated sample/OD of untreated sample) × 100. Macrophage cell viability following nanostructure treatment was also determined using Zombie Aqua fixable cell viability dye. Following differentiation, cells were plated at 2.5 × 10^5 cells mL^{-1} (200 µL, 50,000 cells $well^{-1}$) in 96-well tissue culture treated plates in DMEM cell culture media. RAW 264.7 cells received matched volume treatments of either PBS or $PPSU_{20}$ nanostructures (bundle or vesicular morphology with concentrations of 1, 0.5, 0.25 and 0.125 mg mL^{-1}). After incubation for 24 h, cells were collected and transferred to 1.2 mL microtiter tubes prior to staining with Zombie Aqua fixable viability dye (Biolegend) for 15 min. Cells were washed with cell staining buffer and fixed with intracellular (IC) cell fixation buffer (Biosciences). Flow cytometry data was acquired on an LSR Fortessa analyzer (BD Biosciences) and analyzed using FlowJo.

Cellular uptake studies. RAW 264.7 cells (2.5 × 10^5 cells mL^{-1}, 400 µL) were seeded in each well of a 48-well plate and left overnight in the incubator for adherence. The adhered cells were treated with 0.25 mg mL^{-1} of FITC-dextran-loaded $PPSU_{20}$ nanogels (bundle or vesicular morphology) and incubated for 1 h or 4 h. Cells were washed two times with PBS and incubated with 50 µL Zombie Aqua (1:100) fixable cell viability dye diluted in cell staining buffer for 15 min at 4 °C. Then the cells were washed with 500 µL PBS, resuspended in cell staining buffer and immediately analyzed using a BD Fortessa flow cytometer. The cellular uptake was measured as % of FITC positive cells.

Analysis/imaging of cells. RAW 264.7 cells (1 × 10^5 cells mL^{-1}, 300 µL) were seeded in each well of an 8-well Chamber slide (Thermo Fischer Scientific) and left overnight in the incubator for adherence. The adhered cells were treated with 0.25 mg mL^{-1} of FITC-dextran-loaded $PPSU_{20}$ nanostructures (bundle or vesicular morphology). After incubation for 4 h, cells were washed two times with PBS and incubated with LysoTracker™ Red DND-99 (1:5000 dilution, 300 µL

DMEM) for 30 min. Then the cells were washed twice with PBS, added with 300 μL PBS and incubated with NucBlue™ Live ReadyProbes™ Reagent (nuclei stain, 1 drop) for 15 min in the dark. Images were acquired on a Leica TCS SP8 confocal microscope with a 63 × oil immersion objective.

Investigation of cellular uptake mechanisms. RAW 264.7 macrophages were pre-treated with either cytochalasin D (5 μM; Cayman Chemical) for 2 h to inhibit macropinocytosis, or chlorpromazine (100 μM, Chlorpromazine HCl, Sigma Aldrich) for 30 min to inhibit clathrin-dependent endocytosis. PBS-treated cells were included as a control in these experiments. After this pre-treatment period, cells were incubated with 0.25 mg mL^{-1} of FITC-dextran-loaded $PPSU_{20}$ vesicular nanogels or nanobundles, and flow cytometry was performed. The median fluorescence intensity (MFI) was used to quantify the cellular internalization of $PPSU_{20}$ nanogels. Sidak's multiple comparisons test was used to determine whether inhibitor pre-treatment significantly reduced the cellular uptake of $PPSU_{20}$ nanostructures compared to PBS-treated control. In these experiments, a statistically significant reduction in nanostructure uptake indicates the inhibited endocytosis pathway contributes to internalization.

Supplementary Figures

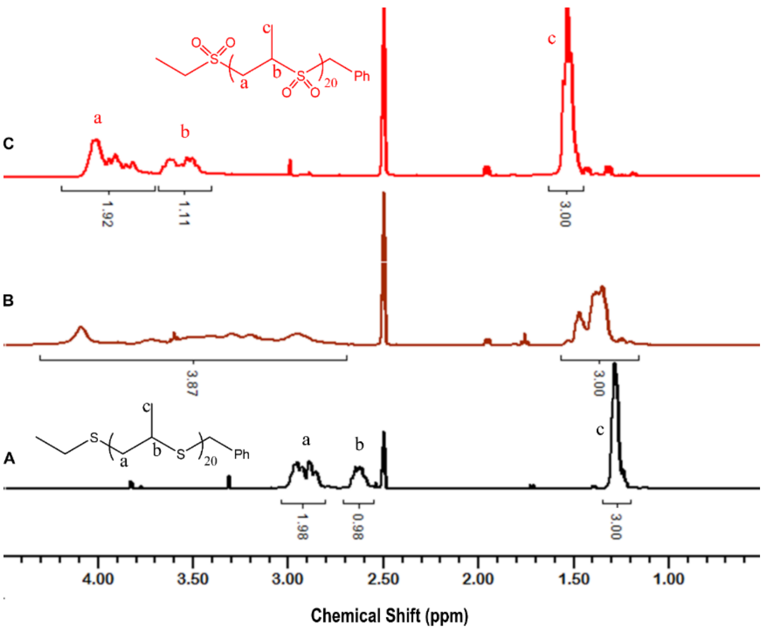

Supplementary Figure 1. ^{1}H NMR spectra in DMSO-d$_6$. (A) PPS$_{20}$, (B) incompletely oxidized product of PPS$_{20}$ (random copolymers of sulfoxides and sulfones), and (C) PPSU$_{20}$.

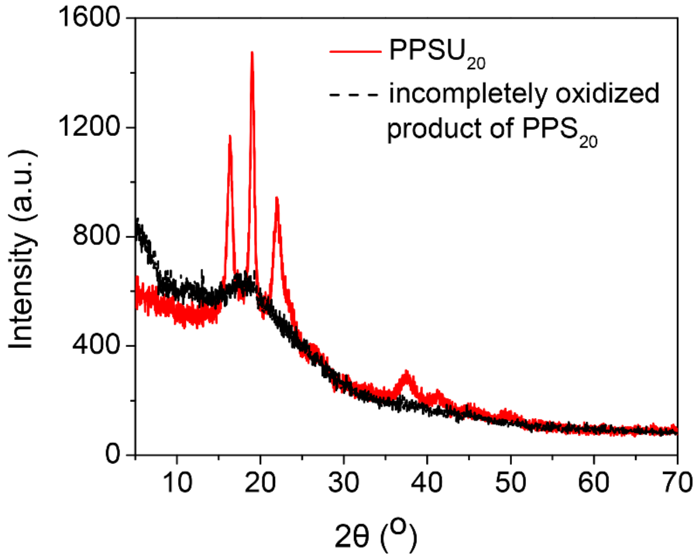

Supplementary Figure 2. WAXD patterns for $PPSU_{20}$ powders and incompletely oxidized product of PPS_{20} (sulfoxide/sulfone mixtures).

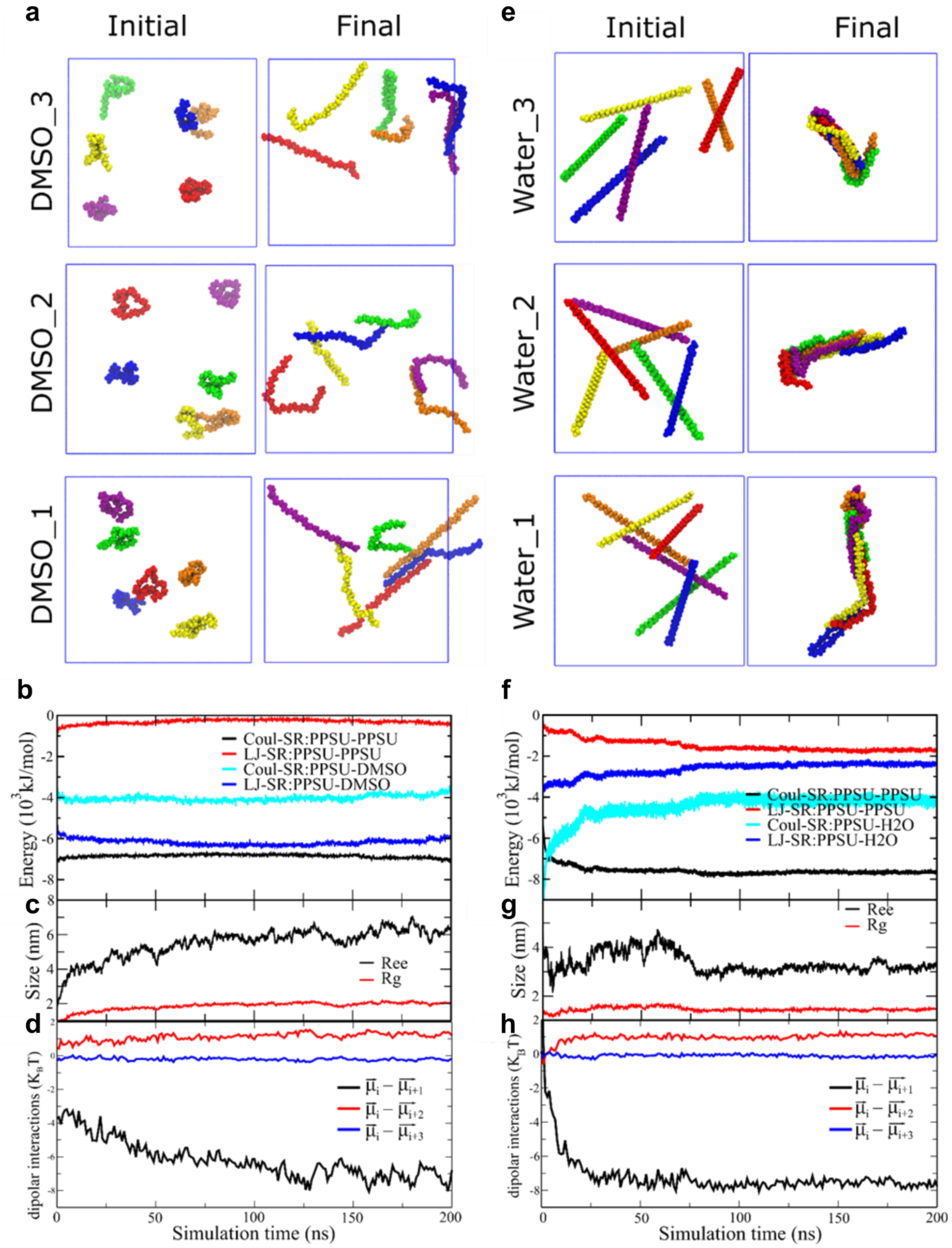

Supplementary Figure 3. Atomistic simulation snapshots and convergence of $PPSU_{20}$. (a) The initially coiled $PPSU_{20}$ chains turned into extended conformation in DMSO simulations. **(b)** The short-range Coulombic (Coul-SR) and LJ (LJ-SR) interactions in DMSO simulations. **(c)** The sizes

of the $PPSU_{20}$ chains described via the end-to-end distance (R_{ee}) and the radius of gyration (R_g) in DMSO simulations. **(d)** The dipolar interactions between PPSU monomers in DMSO simulations. (**e**) The initially extended $PPSU_{20}$ chains collapsed and aggregated in water simulations. **(f)** The Coul-SR and LJ-SR interactions in water simulations. **(g)** The R_{ee} and R_g of $PPSU_{20}$ chains in water simulations. **(h)** The dipolar interactions between PPSU monomers in water simulations. Three parallel simulations were performed for both solvents (6 chains, 12.5 mg mL^{-1}). Hydrogen atoms on $PPSU_{20}$ and solvent molecules are omitted for clarity. The six $PPSU_{20}$ chains are colored differently. The blue solid line denotes the simulation box. The Coul-SR and LJ-SR interactions were calculated up to a cutoff distance of 1.2 nm. The R_{ee} and R_g were calculated using the sulfur atoms on PPSU. The dipolar interactions were calculated between PPSU monomers which are covalently connected ($\overrightarrow{\mu_i} - \overrightarrow{\mu_{i+1}}$), or neighbors of $\overrightarrow{\mu_i} - \overrightarrow{\mu_{i+2}}$, or $\overrightarrow{\mu_i} - \overrightarrow{\mu_{i+3}}$. All the calculations supported that the simulations were roughly converged after around 100 ns.

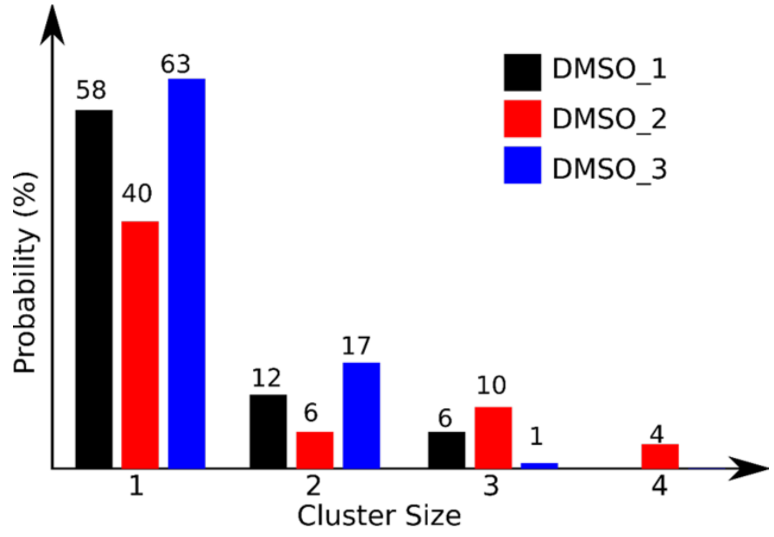

Supplementary Figure 4. Distribution of $PPSU_{20}$ clusters in the DMSO simulations. $PPSU_{20}$ chains are viewed as clusters if the distance of any inter-chain sulfur atoms is less than 0.67 nm (the first minimum in the radial distribution function in Supplementary Figure 8). A maximum probability occurs at the cluster size of 1 supports that the PPSU chains are dispersed in DMSO solvent[11]. The GROMACS program *gmx clustersize* was employed for the calculations.

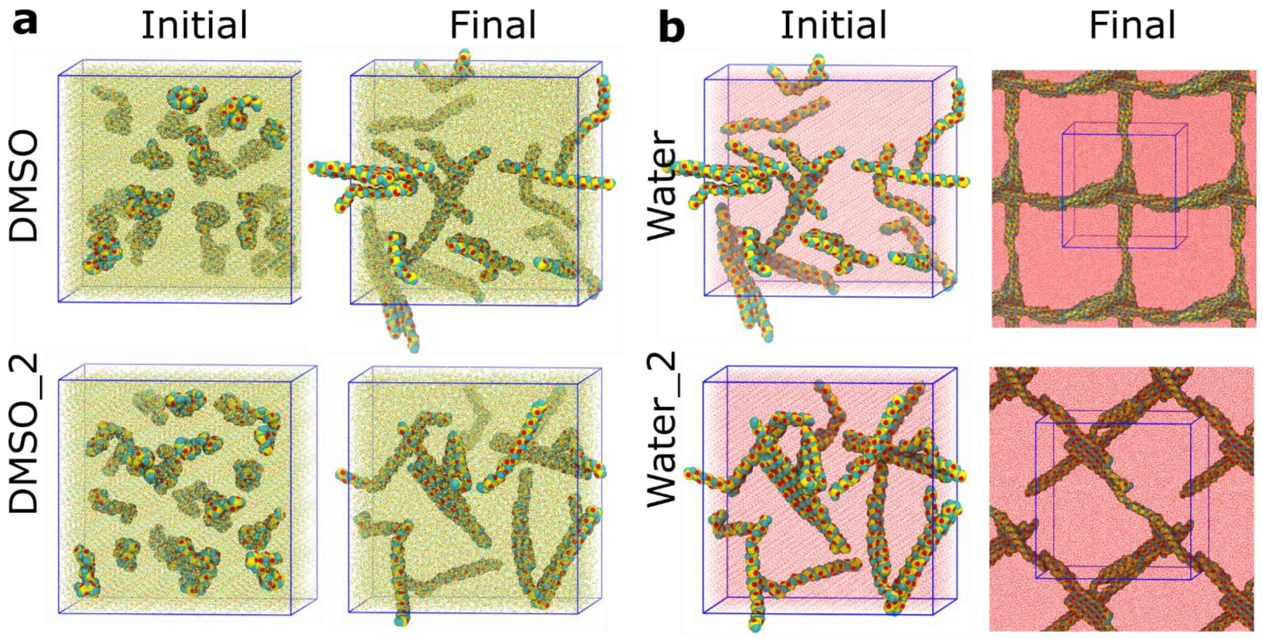

Supplementary Figure 5. Atomistic simulation snapshots of $PPSU_{20}$ in DMSO and water. (**a**) The initially coiled $PPSU_{20}$ chains turned into extended conformation in DMSO. (**b**) Under the application of solvent replacement from DMSO to water, network structure of $PPSU_{20}$ formed due to inter-chain associations. Two parallel simulations were performed for both systems (22 chains, 25 mg mL^{-1}).

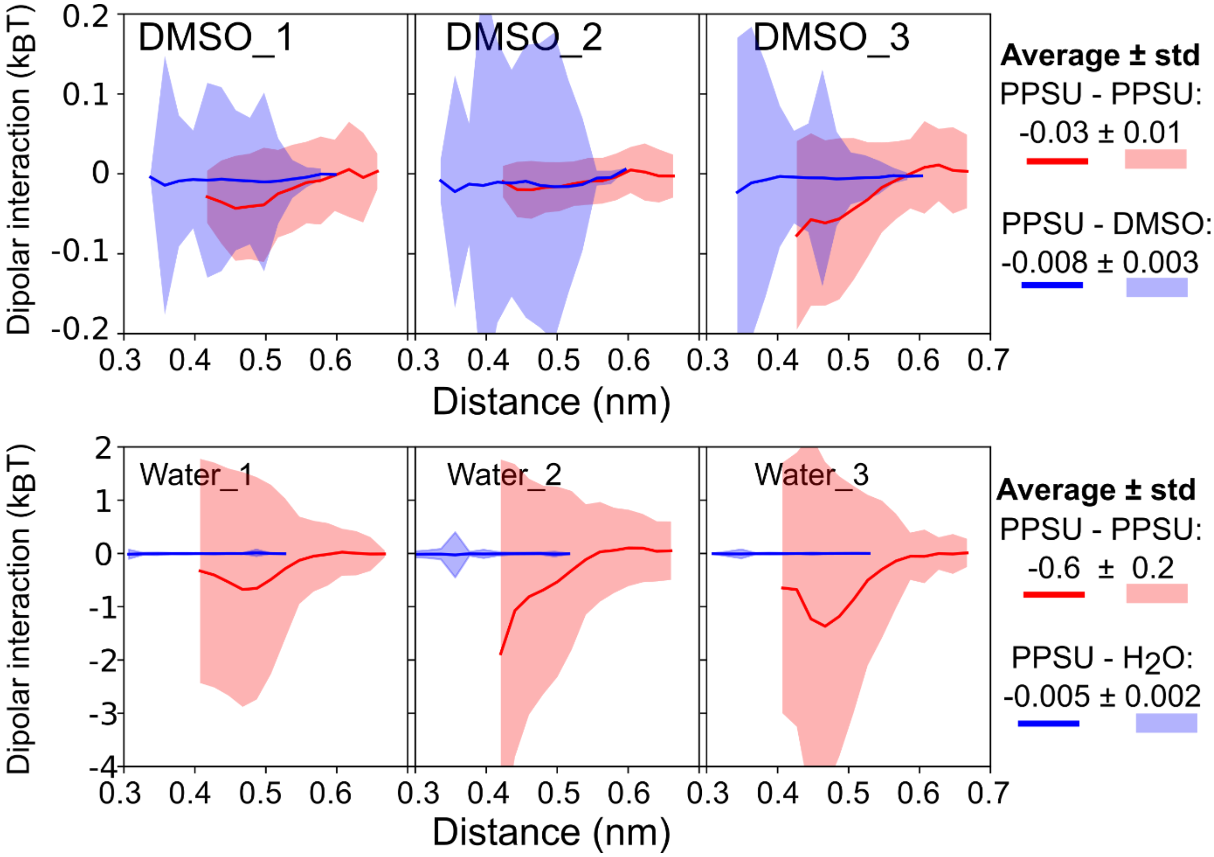

Supplementary Figure 6. Calculation of dipolar energy per dipole-dipole pair. The solid lines stand for the average value as a function of the distance, which is based on the sulfur atoms of PPSU and DMSO, and the oxygen atoms of water. The shadow regions describe the standard deviation of corresponding dipolar energies. Listed in the inset on the right are the average dipolar energies and the standard deviations, which are calculated from the three parallel simulations for the whole distance range. Note that for the S(PPSU)-S(PPSU) calculations all the intramolecular interactions within 5 consecutive repeating units were excluded based on the calculated persistence of 4.4 repeating units in water (Supplementary Table 3).

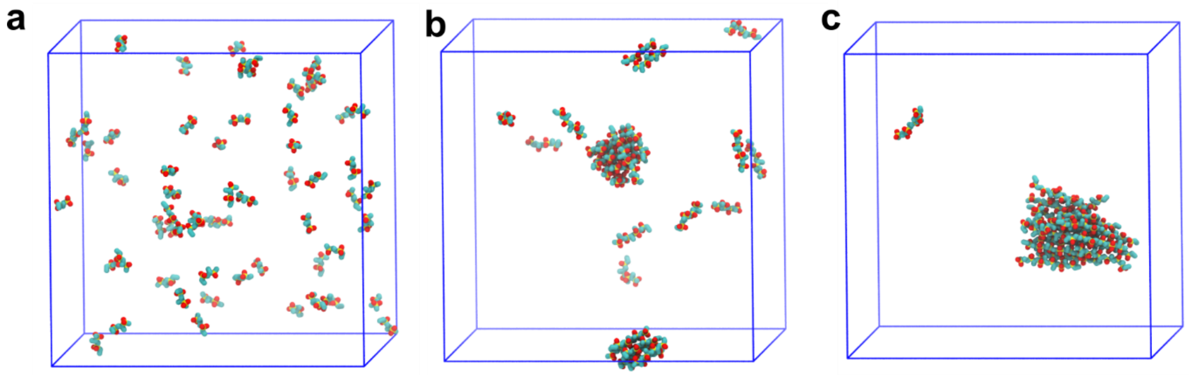

Supplementary Figure 7. Final atomistic simulation snapshots of propylene sulfone oligomers in water. Intermolecular associations increase as degree of polymerization increase from dimers (**a**) to tetramers (**b**) and hexamers (**c**).

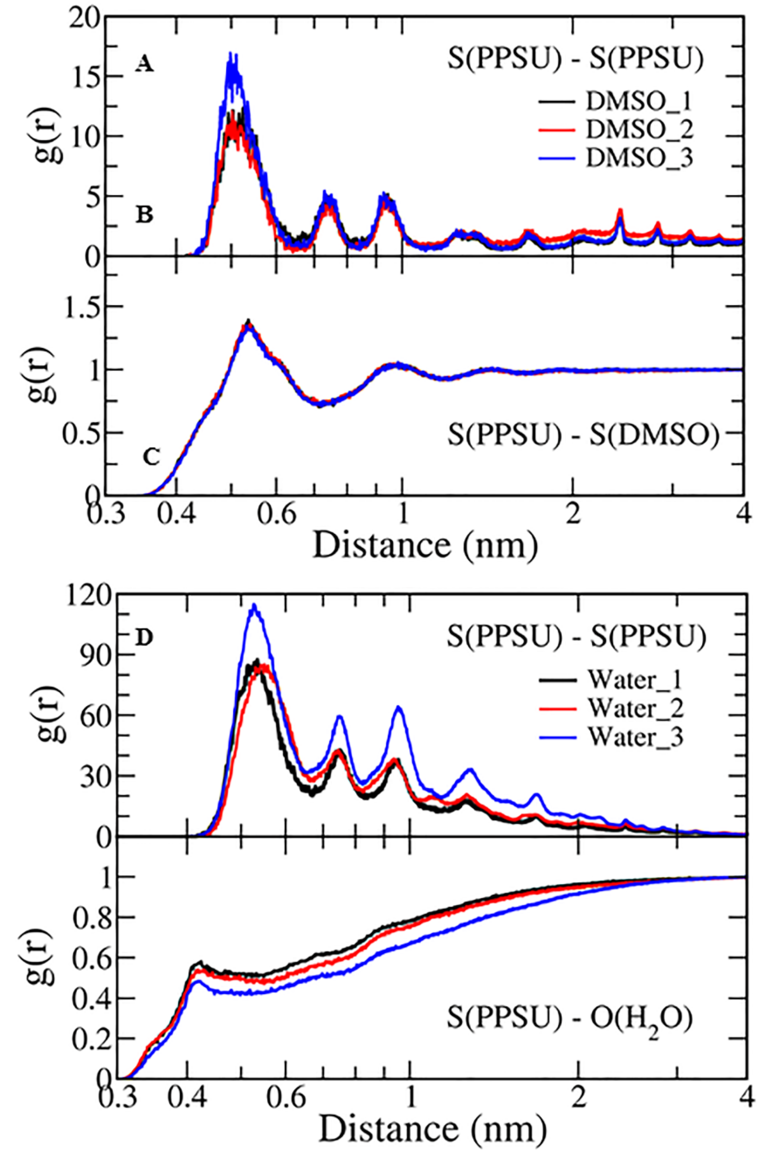

Supplementary Figure 8. Radial distribution functions (RDFs, g(r)) in the (A, B) DMSO and (C, D) aqueous systems. Results are obtained from three parallel all-atom simulations. In the calculations, all the intra-molecular S(PPSU)-S(PPSU) correlations within 5 repeat units were excluded based on persistence length of 4.4 monomers in water (Supplementary Table 3). The RDFs between the sulfur atoms on $PPSU_{20}$ in the aqueous solutions supporting a crystalline structure for $PPSU_{20}$.

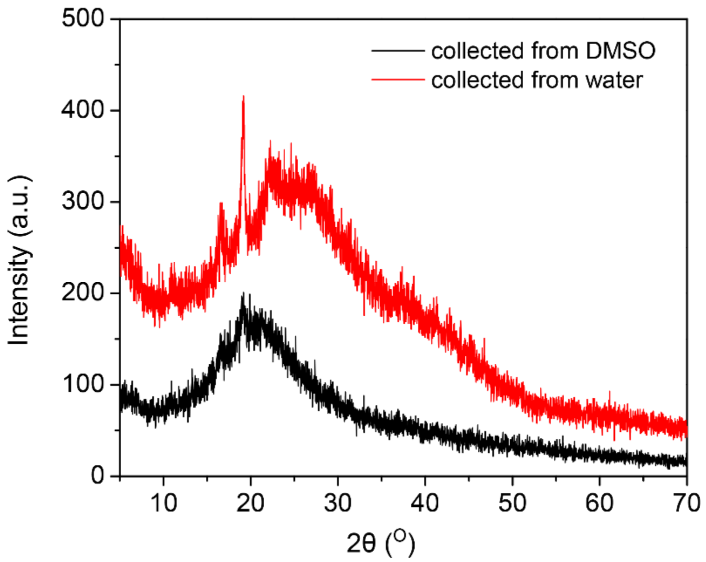

Supplementary Figure 9. WAXD patterns for $PPSU_{20}$ precipitates collected from DMSO or water. The fluffy precipitates were obtained by exposing 200 μL of DMSO solution of $PPSU_{20}$ (25 mg mL^{-1}) to humidity in air for 110 days. After centrifugation, part of the precipitates was applied for WAXD directly, and the other precipitates was dispersed in water and recollected for WAXD.

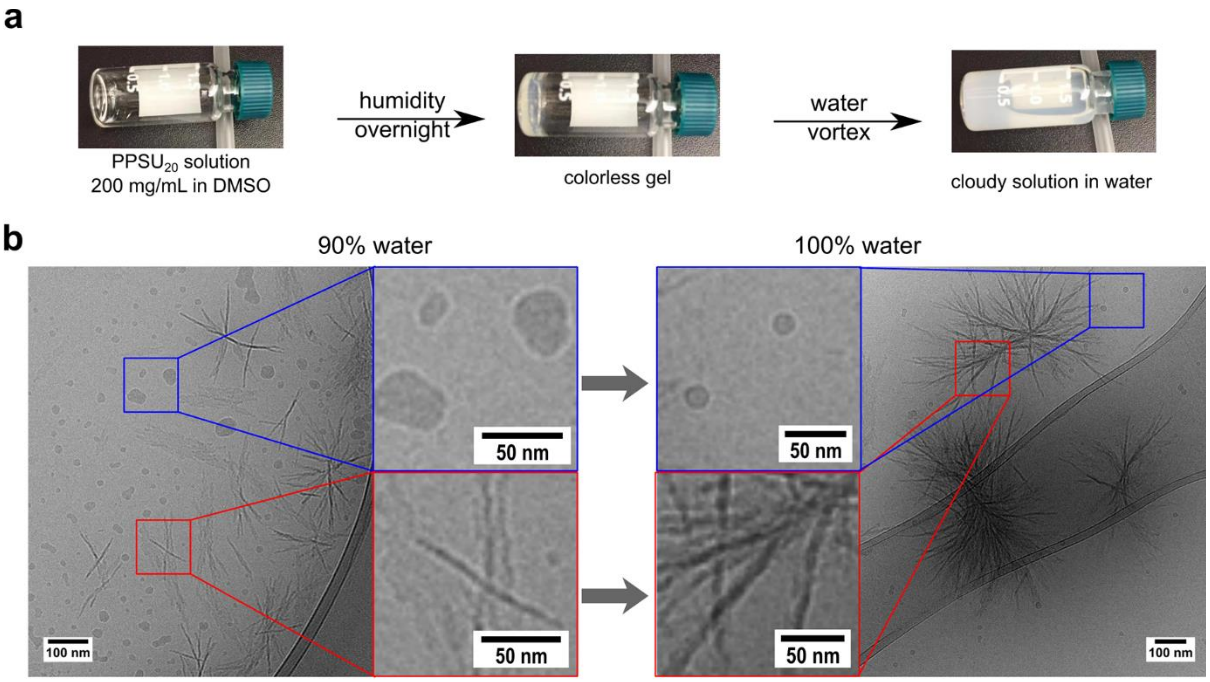

Supplementary Figure 10. Quick hydration leading to large scale spatial redistribution of PPSU$_{20}$ chains on network. (**a**) Exposure and aging of a highly concentrated DMSO solution of PPSU$_{20}$ in air leads to a colorless gel. Thoroughly mixing of the gel with water results in a cloudy solution. (**b**) Cryo-TEM images of the cloudy solution showing PPSU$_{20}$ nanostructures in a water-DMSO system (1/9, V/V) and in 100% water. Non-uniform aggregates including ribbons formed in 90% water, which reorganized into bundles and vesicular nanogels after dialysis to remove residual DMSO.

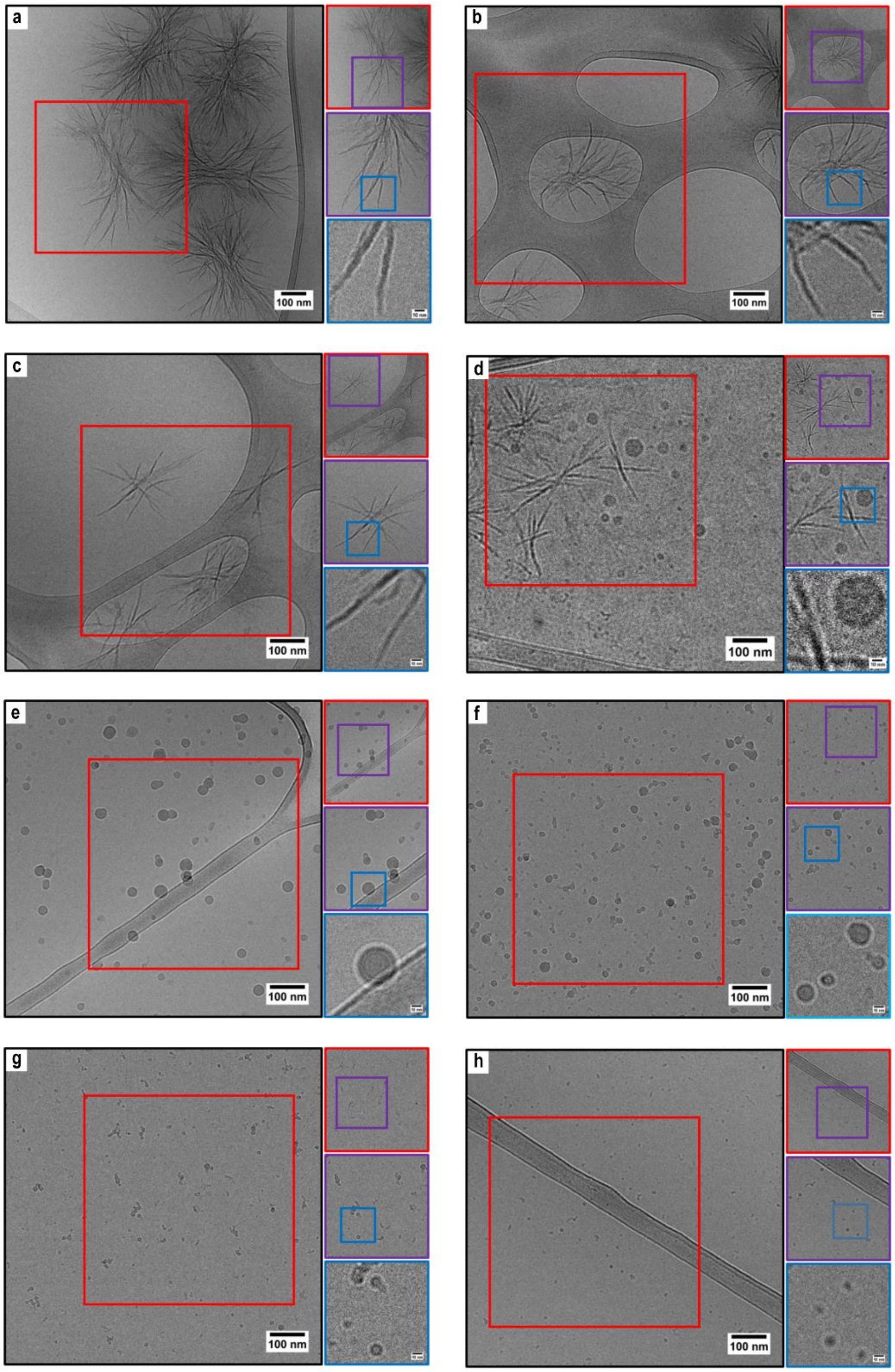

Supplementary Figure 11. CryoTEM of $PPSU_{20}$ nanogels in water. The nanogels were prepared by stepwise hydration of DMSO solutions of $PPSU_{20}$ (25 mg mL^{-1}, 200 µL) with 400 µL of water (see Fig. 2a): (**a**) 5 µL *per* step, 80 steps; (**b**) 10 µL *per* step, 40 steps; (**c**) 20 µL *per* step, 20 steps; (**d**) 50 µL *per* step, 8 steps; (**e**) 100 µL *per* step, 4 steps; (**f**) 200 µL *per* step, 2 steps; (**g**) 400 µL *per* step, 1 step; (**h**) 800 µL *per* step, 1 step. Images were obtained by CryoEM after removal of DMSO. Size of selection: red (600 nm × 600 nm), purple (300 nm × 300 nm), blue (100 nm × 100 nm).

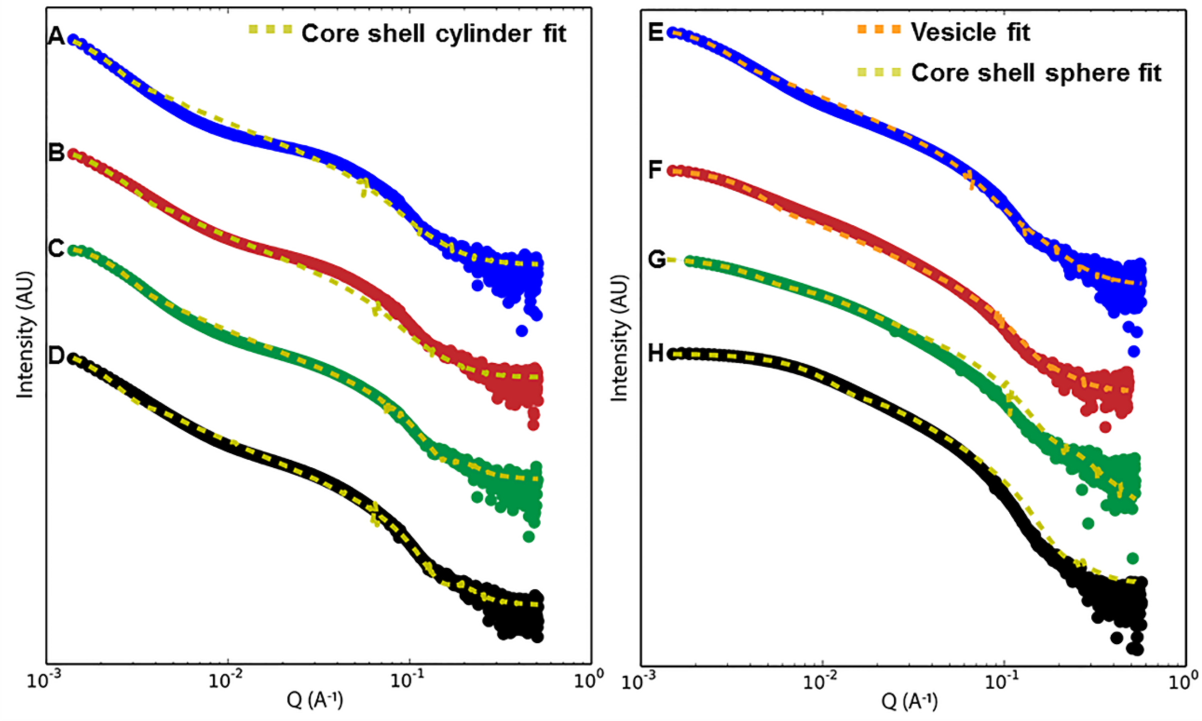

Supplementary Figure 12. SAXS of PPSU$_{20}$ nanogels in water. The samples were prepared by stepwise hydration of DMSO solutions of PPSU$_{20}$ (25 mg mL^{-1}, 200 µL) with 400 µL of water (see Fig. 2a), followed by dialysis in water to remove DMSO. (**A**) 5 µL *per* step, 80 steps; (**B**) 10 µL *per* step, 40 steps; (**C**) 20 µL *per* step, 20 steps; (**D**) 50 µL *per* step, 8 steps; (**E**) 100 µL *per* step, 4 steps; (**F**) 200 µL *per* step, 2 steps; (**G**) 400 µL *per* step, 1 step; (**H**) 800 µL *per* step, 1 step.

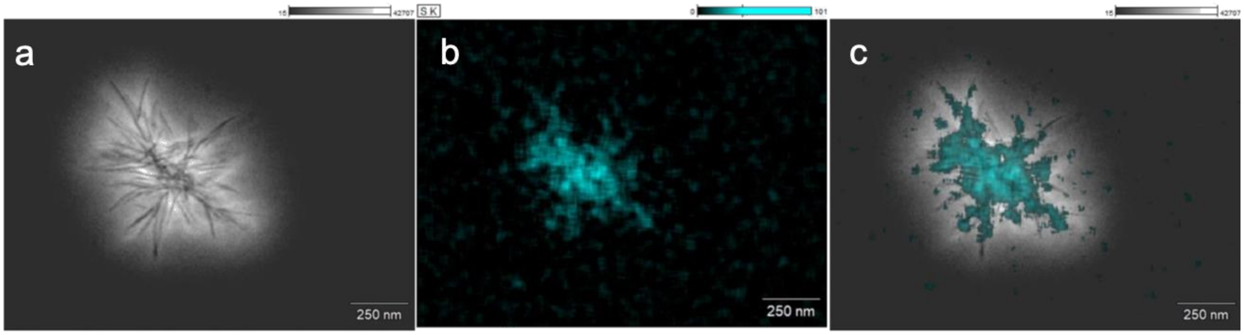

Supplementary Figure 13. Confirming the formation of nanobundle by negative-stained transmission electron microscopy and energy dispersive X-ray spectroscopy. (**a**) HAADF STEM image. (**b**) The corresponding sulfur EDS map on the right. (**c**) Overlay of base image and sulfur map. The samples correspond to sample C in Supplementary Figure 11.

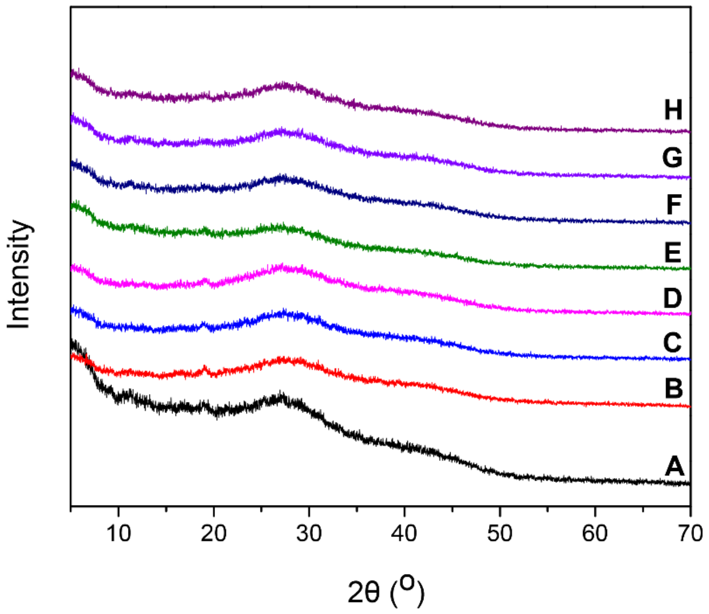

Supplementary Figure 14. WAXD patterns for $PPSU_{20}$ nanostructures. The samples were prepared by stepwise hydration of DMSO solutions of $PPSU_{20}$ (25 mg mL^{-1}, 200 µL) with 400 µL of water (see Fig. 2a), followed by dialysis in water to remove DMSO. (**A**) 5 µL *per* step, 80 steps; (**B**) 10 µL *per* step, 40 steps; (**C**) 20 µL *per* step, 20 steps; (**D**) 50 µL *per* step, 8 steps; (**E**) 100 µL *per* step, 4 steps; (**F**) 200 µL *per* step, 2 steps; (**G**) 400 µL *per* step, 1 step; (**H**) 800 µL *per* step, 1 step.

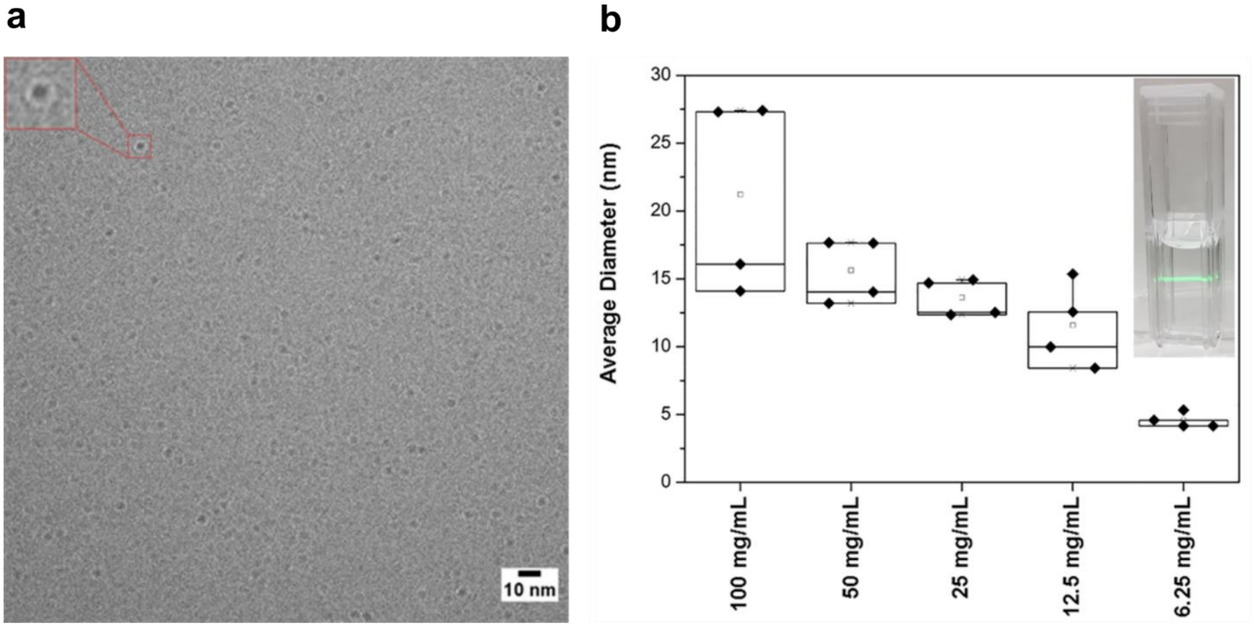

Supplementary Figure 15. Quick hydration of DMSO solution of $PPSU_{20}$ leading to tiny spherical nanogels. (**a**) CryoTEM image showing the formation of tiny spherical nanogels by quick hydration of 50 μL $PPSU_{20}$ solution (25 mg mL^{-1} in DMSO) with 1 mL of water (scale bar = 10 nm, the red selection = 10 nm × 10 nm). (**b**) Number-average diameters determined by DLS for micellar solutions prepared from quick hydration of 50 μL $PPSU_{20}$ solutions (different concentrations in DMSO) by the using of 1 mL water. Inset is a clear solution prepared by mixing a 25 mg mL^{-1} DMSO solution of $PPSU_{20}$ with 1 mL of water, showing strong Tyndall effect.

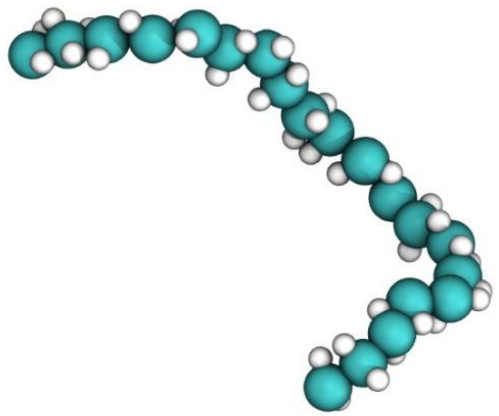

Supplementary Figure 16. Coarse-grained model of a $PPSU_{20}$ chain. Each chain is modeled by a linear bead-spring chain consisting of N = 20 monomers. Each monomer carries three point charges: a positive charge in the backbone for the S atom (green sphere) and two negative charges for the O atoms (white spheres). The relative positions of the three point charges are maintained by harmonic springs between the S-O bonds and constraining the O-S-O angle. The non-bonded interactions between the coarse-grained monomers includes the excluded volume interaction and electrostatics.

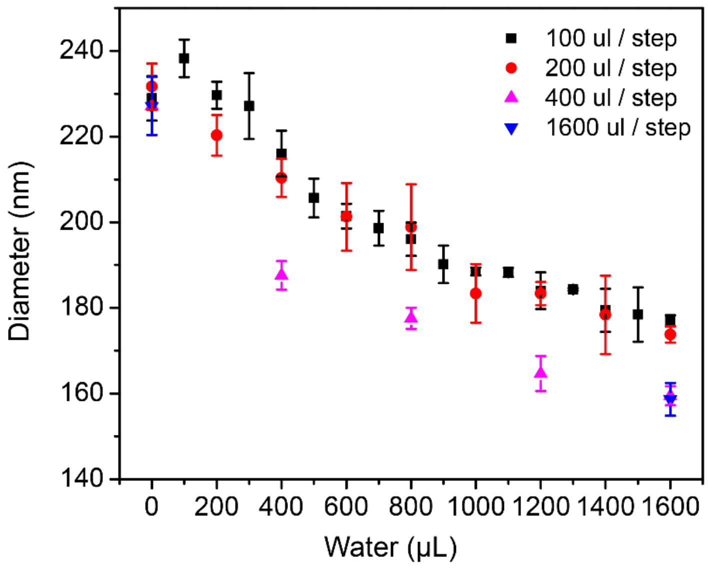

Supplementary Figure 17. DLS measurements showing sizes decreasing of $PPSU_{20}$ assemblies upon stepwise hydration. The sizes of assemblies were assessed following the initial mixing of 330 μL of DMSO solution of PPSU20 (25 mg mL^{-1}) with 660 μL water and subsequent stepwise hydration. Error bars represent the standard deviation from three parallel experiments.

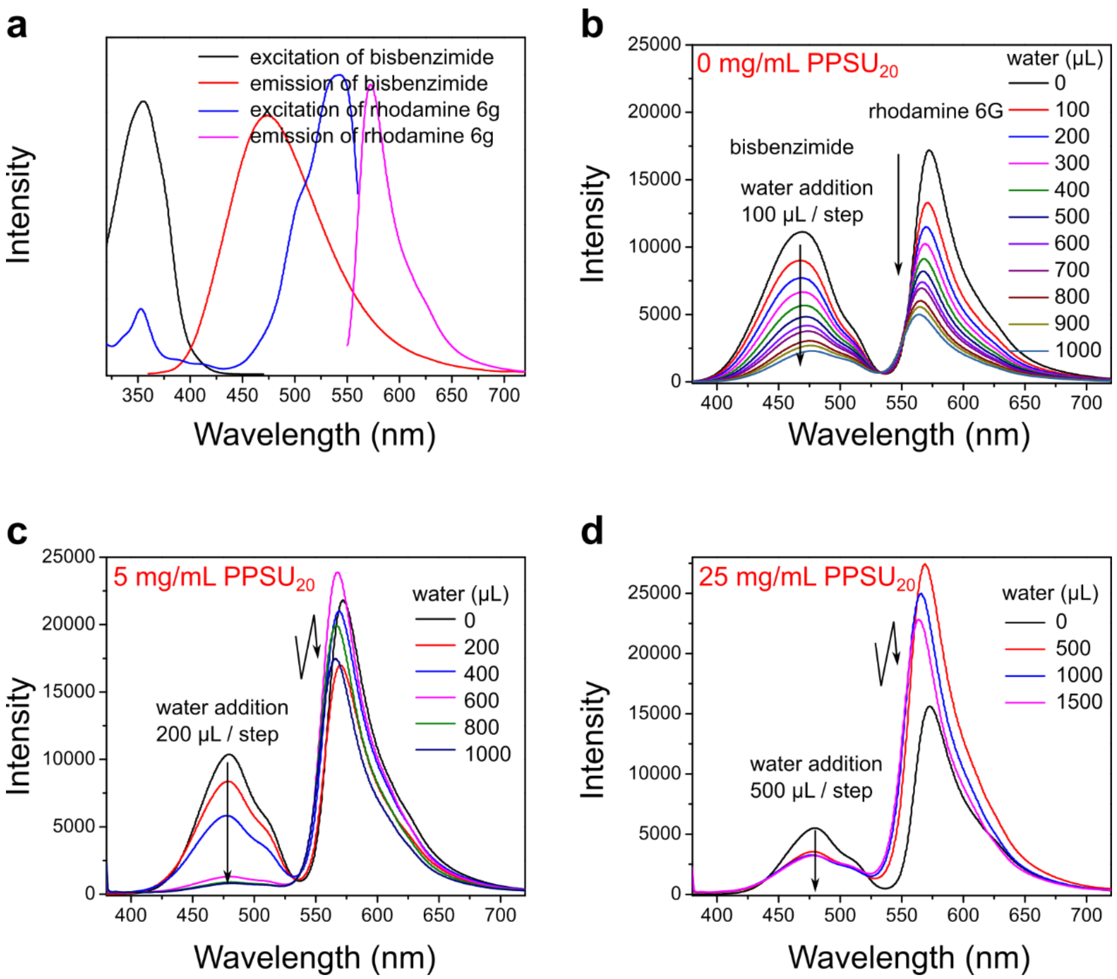

Supplementary Figure 18. Förster resonance energy transfer (FRET) from bisbenzimide to rhodamine 6G. (**a**) Excitation and emission spectra of bisbenzimide to rhodamine 6g in DMSO. (**b-d**) Fluorescence titration (E_x = 375 nm) experiments of 1 mL DMSO solution of bisbenzimide (0.01 mg/mL), rhodamine 6G (0.01 mg/mL), and $PPSU_{20}$ (0, 5, 25 mg mL^{-1}) with water. (**b**) Without $PPSU_{20}$, water dilutes the solution and decreases fluorescence intensity for both bisbenzimide and rhodamine 6g. (**c-d**) In the presence of $PPSU_{20}$, obvious FRET signals from bisbenzimide to rhodamine 6g are induced upon stepwise hydration. The arrows denote the change of fluorescence intensity.

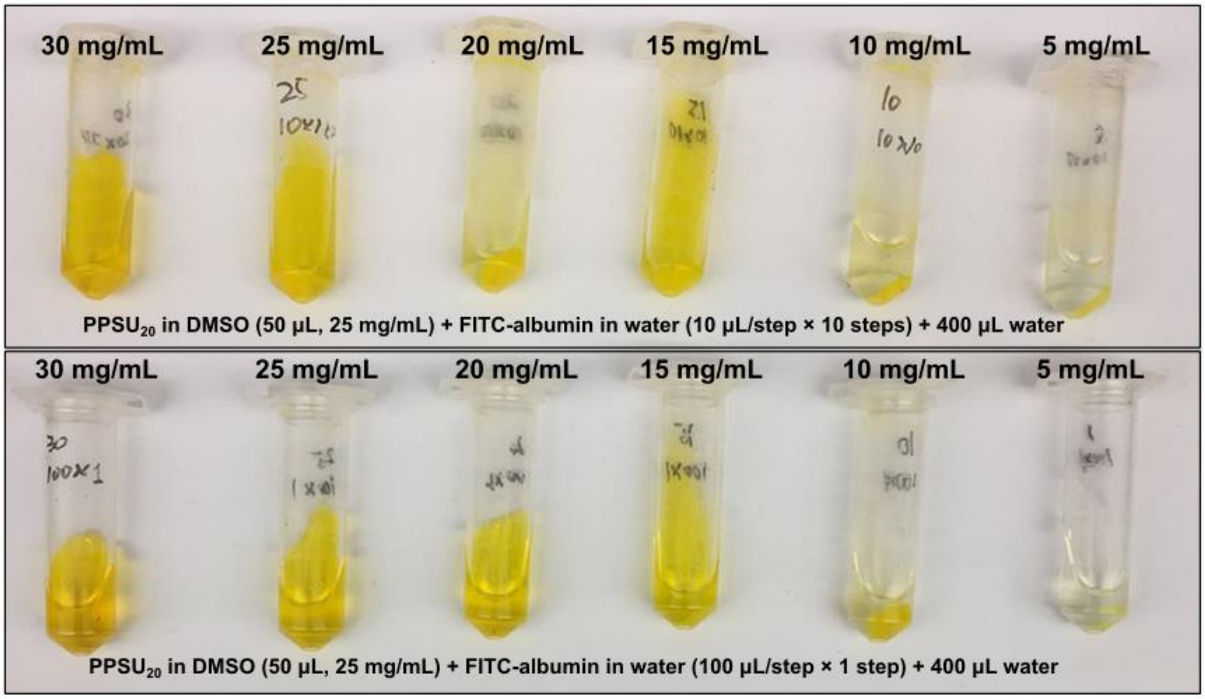

Supplementary Figure 19. Loading FITC-albumin during $PPSU_{20}$ assembly. 50 µL of $PPSU_{20}$ solutions (25 mg mL^{-1} in DMSO) were added stepwise with 100 µL of aqueous FITC-albumin solutions (30 mg mL^{-1} to 5 mg mL^{-1}) and then one-time with 400 µL of water. Each step was followed by vortexing to thoroughly mix the samples. Photos were taken after centrifugation (16,000 g, 10 min).

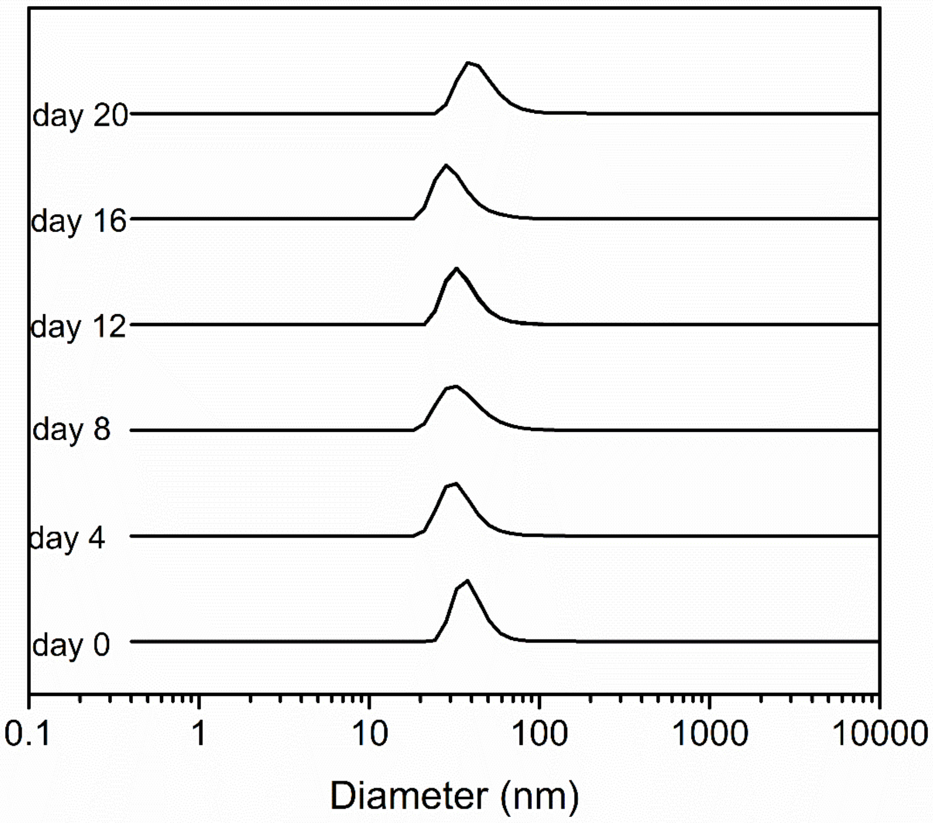

Supplementary Figure 20. Stability study of $PPSU_{20}$ vesicular nanogels in water over time. Representative DLS plots giving the size distribution of $PPSU_{20}$ vesicular nanogels in the range of 20–100 nm without occurrence of nanogel aggregation within 20 days at room temperature.

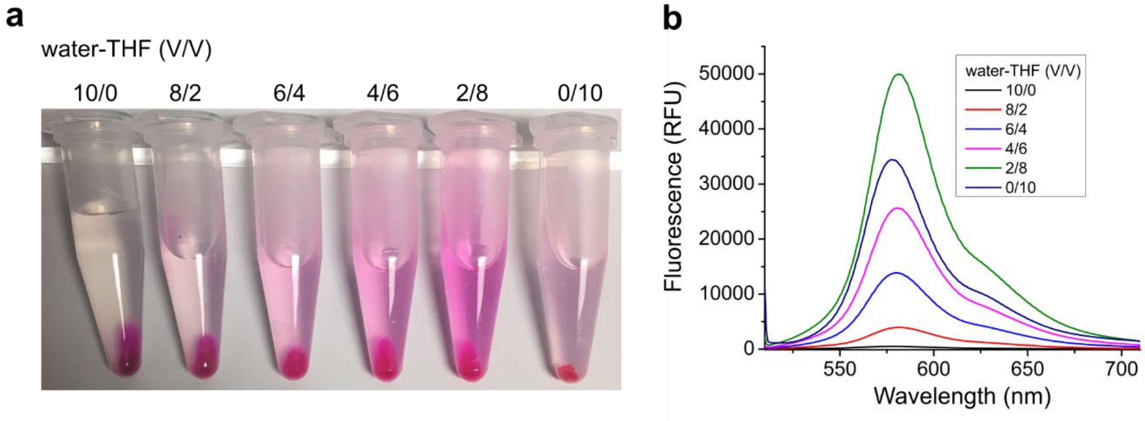

Supplementary Figure 21. Leakage of rhodamine B (Rh B) from PPSU$_{20}$ vesicular nanogels in tetrahydrofuran (THF)-water mixed systems. (**a**) Photos of Rh B-loaded PPSU$_{20}$ vesicular nanogels in THF-water mixed solvents after 3-day incubation (room temperature) and centrifugation (16,000 g, 10 min). (**b**) Fluorescence spectra of the corresponding supernatants in (**a**). The supernatants (0.5 mL) were diluted with THF and water to 2 mL of water-THF (8/2, V/V) mixed solvent for fluorescence measurements, E_x = 500 nm.

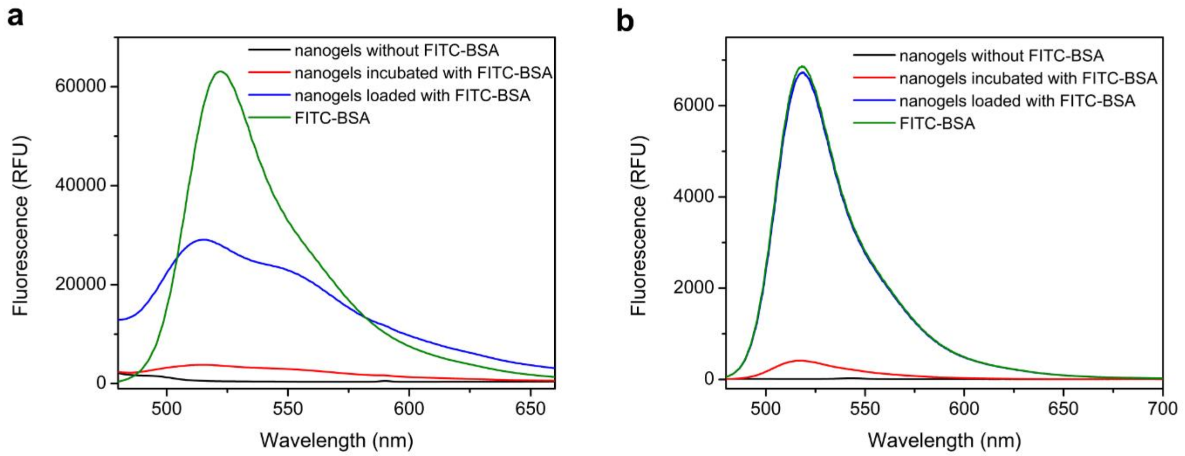

Supplementary Figure 22. Fluorescence spectra (E_x = 460 nm) verifying negligible adsorption of FITC-BSA by preformed $PPSU_{20}$ vesicles. (**a**) Fluorescence spectra of blank vesicles, FITC-BSA-adsorbed vesicles, FITC-BSA-loaded vesicles, and for comparison FITC-BSA (0.1 mg mL^{-1}) in water. FITC-BSA molecules were highly concentrated after encapsulation in nanogels and the fluorescence was partially quenched in water. (**b**) Fluorescence spectra of blank vesicles, FITC-BSA-adsorbed vesicles, FITC-BSA-loaded vesicles, and for comparison FITC-BSA (0.01 mg mL^{-1}) in aqueous NaOH (0.2 N) solution. Fluorescence of FITC-BSA recovered in aqueous NaOH solution due to the breaking of vesicular nanogels. Fluorescence quantitative analysis (E_m = 518 nm) confirmed that while the encapsulation efficiency was 97.8%, the adsorption efficiency of FITC-BSA was 6.0%. E_x = 460 nm.

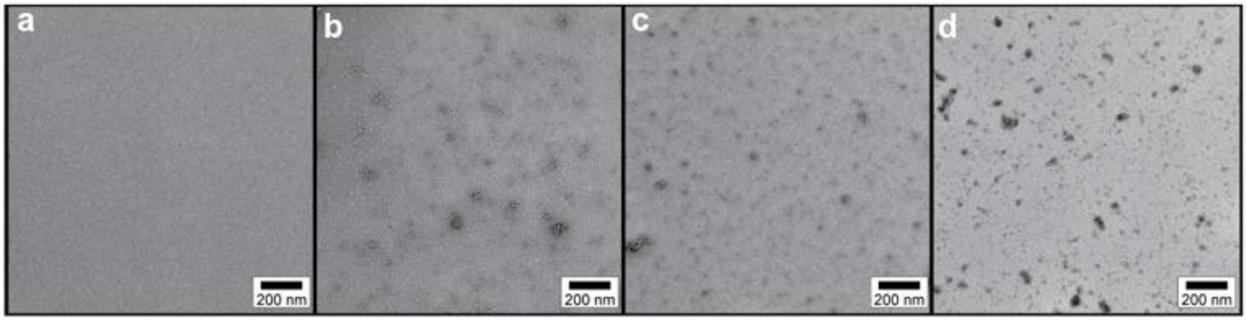

Supplementary Figure 23. Transmission electron microscopy of aqueous FITC-BSA stained with 1.5% uranyl formate as a control. The concentrations of the protein samples (**a**-**d**) were 0, 0.1, 1.0, and 10 mg mL^{-1}, respectively.

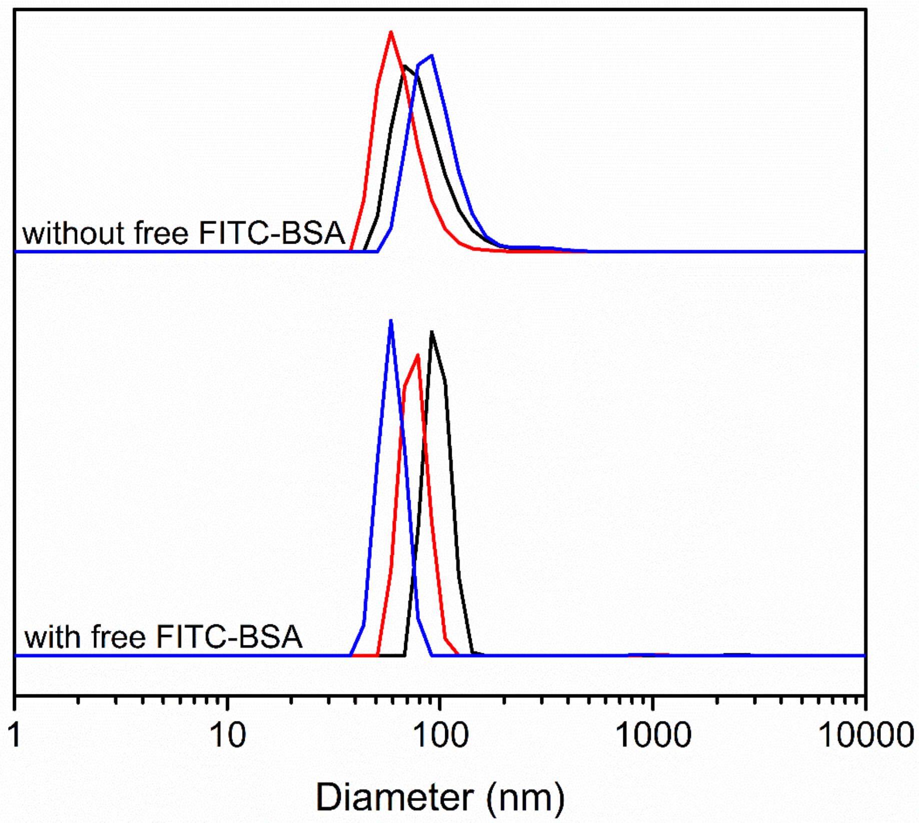

Supplementary Figure 24. DLS plots showing the size distribution of $PPSU_{20}$ vesicular nanogels that were loaded with FITC-BSA at a saturating concentration (10 mg mL^{-1}, 160% w/w protein/PPSU). DLS measurements were performed for the samples with or without removing free FITC-BSA by centrifugation. Black, red, and blue DLS plots were obtained from three parallel experiments. No statistically significant differences in average diameter between the two groups of samples were found.

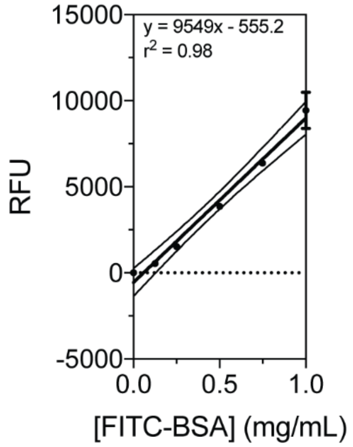

Supplementary Figure 25. Calibration curve of FITC-BSA fluorescence (E_x = 495 nm, E_m = 519 nm) for protein concentration measurement. Fit linear regression model (y = 9549x – 555.2, r^2 = 0.98) was used for concentration measurement. Bands represent the 95% confidence interval. The calibration curve was constructed using 3 replicates.

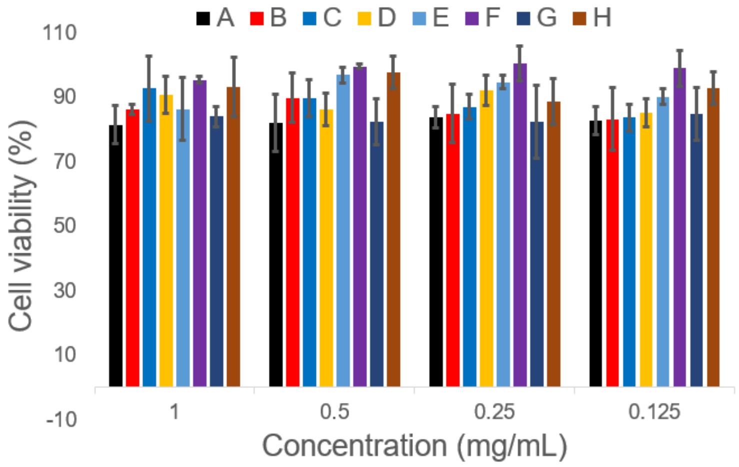

Supplementary Figure 26. MTT assay of $PPSU_{20}$ nanogels on RAW 264.7 cells for 24 h. Samples A-H were prepared by stepwise hydration of DMSO solutions of $PPSU_{20}$ (25 mg mL^{-1}, 200 μL) with 400 μL of water (see Fig. 2a), followed by dialysis in water to remove DMSO. (A) 5 μL *per* step, 80 steps; (B) 10 μL *per* step, 40 steps; (C) 20 μL *per* step, 20 steps; (D) 50 μL *per* step, 8 steps; (E) 100 μL *per* step, 4 steps; (F) 200 μL *per* step, 2 steps; (G) 400 μL *per* step, 1 step; (H) 800 μL *per* step, 1 step. Error bars represent the standard deviation from 4 parallel experiments. No statistically significant difference was found between the experimental materials and the PBS control group.

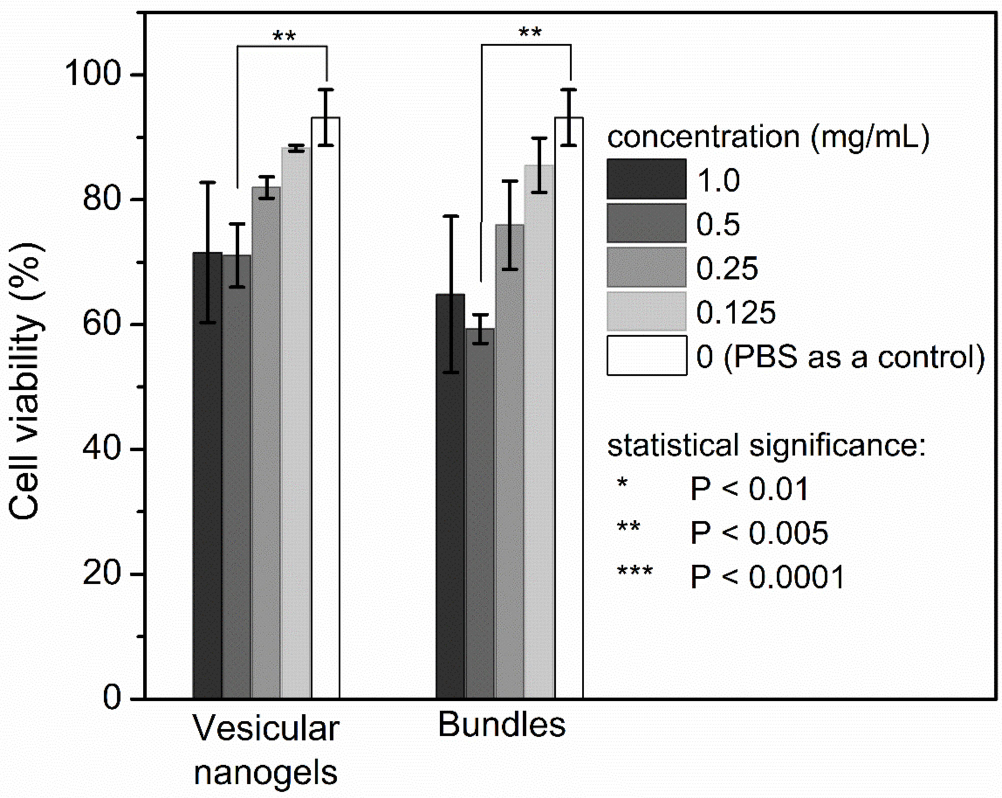

Supplementary Figure 27. Cytotoxicity of $PPSU_{20}$ nanogels. Flow cytometric-based toxicity assessment of $PPSU_{20}$ vesicular nanogels and bundles on RAW 264.7 cells for 24 h. Error bars represent the standard deviation from 3 parallel experiments.

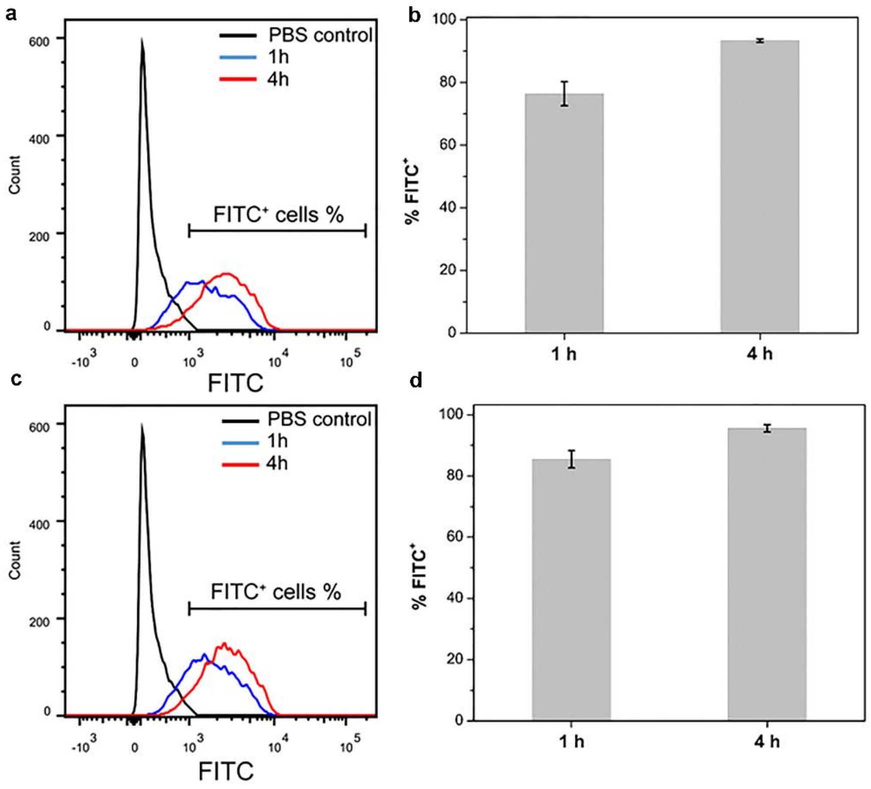

Supplementary Figure 28. Cellular uptake of $PPSU_{20}$ vesicular nanogels (a and b) and bundles (c and d) in RAW 264.7 cells. Nanogels were loaded with FITC-dextran, and incubated with cells for 1 h and 4 h. The percentage of FITC positive (% $FITC^+$) cells is reported to indicate the extent of cellular uptake. Error bars represent the standard deviation from 3 parallel experiments.

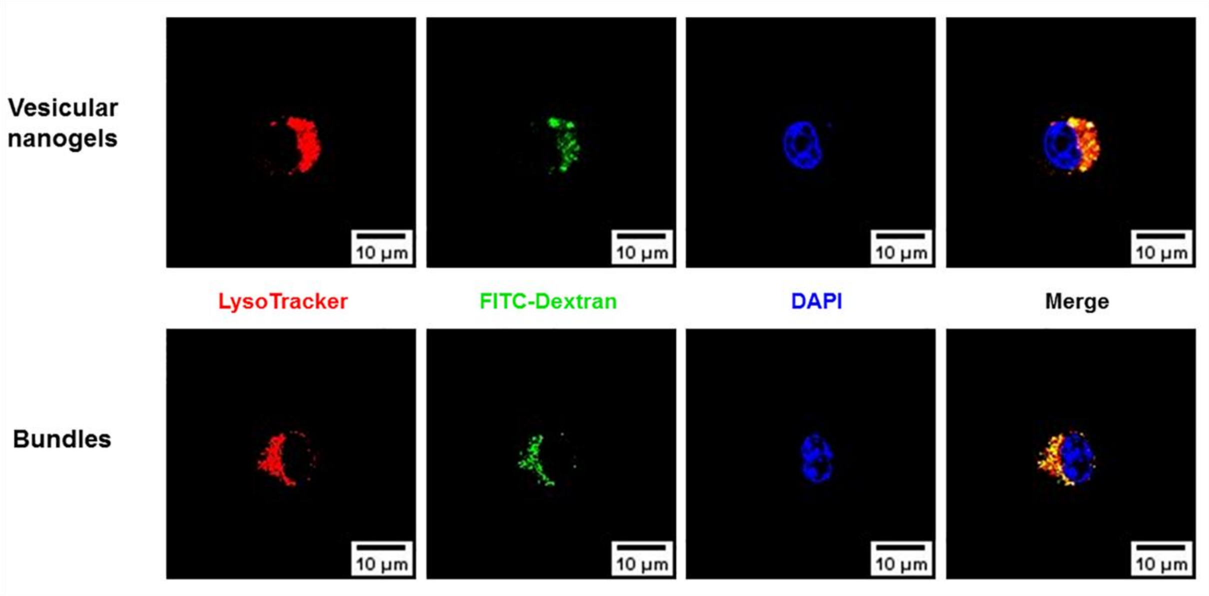

Supplementary Figure 29. Confocal fluorescence images of RAW 264.7 cells. Cells were incubated with FITC-dextran-loaded $PPSU_{20}$ nanogels for 4 h. Nuclei and endosomes/lysosomes were stained by DAPI and LysoTracker Red, respectively. Scale bar = 10 µm.

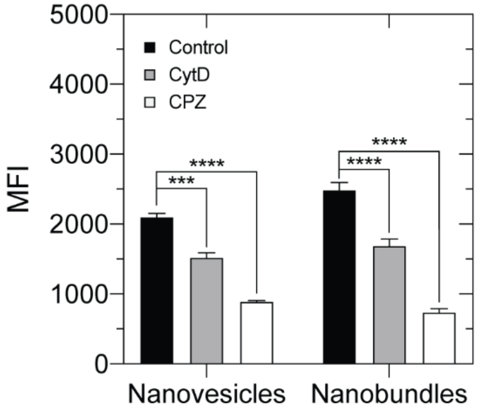

Supplementary Figure 30. Cells internalize $PPSU_{20}$ vesicular nanogels and bundles using macropinocytosis and clathrin-mediated endocytosis mechanisms. Data are presented as the average median fluorescence intensity (MFI) ± s.e.m. (n=3). Statistically significant differences in nanostructure uptake in the absence (PBS pre-treated "Control") or presence of inhibitor (CytD or CPZ) was determined using Sidak's multiple comparisons test (*** p<0.0005, **** p<0.0001).

Supplementary Tables

Supplementary Table 1. Solubility of $PPSU_{20}$ in water and some common organic solvents

**solvent** [a]	**water**	**MeOH**	**AA**	**CAN**	**NMP**	**DMSO**	**DMF**	**DCM**	**Py**	**THF**	**Diox**
**polarity** [b]	**1.000**	**0.762**	**0.648**	**0.460**	**0.458**	**0.444**	**0.386**	**0.309**	**0.302**	**0.207**	**0.164**
crystal [c]	✗	✗	✗	✗	✓ [f]	✓ [g]	✗	✗	✗	✗	✗
solution [d]	C	C	C	C [e]	M	M	M	M	M	M	M

[a] MeOH = methanol, AA = acetic acid, CAN = acetonitrile, NMP = 1-methyl-2-pyrrolidinone, DMSO = dimethylsulfoxide, DMF = dimethylformamide, Py = pyridine, THF = tetrahydrofuran, Diox = dioxane. [b] Relative polarity[12]. [c] Using $PPSU_{20}$ crystals for the solubility tests. ✗ = insoluble, ✓ = soluble. [d] Mixing a DMSO solution of $PPSU_{20}$ (25 mg mL^{-1}) with 9 times the volume of other solvents, then checking the turbidity of the mixtures. C = cloudy, M = miscible. [e] The mixture was clear at the beginning, but became cloudy in 1 hour. [f] Solubility of $PPSU_{20}$ in 1 mL of NMP is ~23 mg. [g] Solubility of $PPSU_{20}$ in 1 mL of DMSO is >250 mg.

Supplementary Table 2. Some Data in the All-atom MD Simulations

Entry [a]	Number of $PPSU_{20}$ chains	Number of solvent molecules	Length of box (nm) [b]	Simulation time (ns) [c]
DMSO_1	6	13893	11.867 ± 0.004	150+50
DMSO_2	6	13896	11.868 ± 0.004	150+50
DMSO_3	6	13894	11.868 ± 0.004	150+50
Water_1	6	56887	11.924 ± 0.006	150+50
Water_2	6	56885	11.924 ± 0.004	150+50
Water_3	6	56877	11.923 ± 0.005	150+50

[a] Three parallel simulations for both DMSO and water solvents. [b] The simulation box length in the production simulations of 50 ns. [c] Time in the equilibration simulation + time in production simulation.

Supplementary Table 3. Results from All-atom MD Simulations[a]

	DMSO				water			
	**Run 1**	**Run 2**	**Run 3**	**Average**	**Run 1**	**Run 2**	**Run 3**	**Average**
R_{ee} [b]	6.2	6.1	5.6	6.0 ± 0.3	4.6	3.2	2.4	3.4 ± 1.0
L_p [c]	9.8	9.6	8.2	9.2 ± 0.7	4.6	4.2	4.3	4.4 ± 0.2

[a] All calculations are based on the sulfur atoms on $PPSU_{20}$. The GROMACS program *gmx polystat* was employed. [b] End-to-end distance, in the unit of nm. R_{ee} = 7.9 nm for a fully extended $PPSU_{20}$ chain. [c] Persistence length, in the unit of repeat units.

Supplementary Table 4. Physicochemical characteristics of $PPSU_{20}$ nanogels in water

Sample [a]	Morphology [b]	Diameter [c] (nm)	Length [c] (nm)	Diameter [d] (nm)	Polydispersity Index [d]	Zeta Potential [d] (mV)
A	bundle	14.8±0.4	435.3±8.1	N/A	N/A	-38.43±0.72
B	bundle	29.2±0.5	344.7±5.4	N/A	N/A	-34.14±0.43
C	bundle	29.4±1.4	324.5±4.0	N/A	N/A	-47.83±0.49
D	bundle/vesicle	18.2±0.3	296.4±1.5	N/A	N/A	-34.67±0.46
E	vesicle	75.2±2.3	N/A	81.7±13.6	0.207±0.043	-28.10±0.70
F	vesicle	53.4±4.0	N/A	37.3±6.9	0.251±0.004	-46.23±0.49
G	vesicle/micelle	33.0±4.9	N/A	25.4±6.0	0.259±0.020	-48.33±1.10
H	micelle	18.6±0.5	N/A	40.9±9.2	0.236±0.017	-45.23±1.40

[a] The samples were prepared by stepwise hydration of DMSO solutions of $PPSU_{20}$ (25 mg mL^{-1}, 200 μL) with 400 μL of water (see Fig. 2a), followed by dialysis in water to remove DMSO. (A) 5 μL *per* step, 80 steps; (B) 10 μL *per* step, 40 steps; (C) 20 μL *per* step, 20 steps; (D) 50 μL *per* step, 8 steps; (E) 100 μL *per* step, 4 steps; (F) 200 μL *per* step, 2 steps; (G) 400 μL *per* step, 1 step; (H) 800 μL *per* step, 1 step. [b] Determined by CryoTEM. [c] Measured by SAXS. [d] Measured by DLS.

Supplementary Table 5. Size stability of $PPSU_{20}$ vesicular nanogels over time in water[a]

	Sample 1		Sample 2		Sample 3	
	Diameter (nm)	PDI	Diameter (nm)	PDI	Diameter (nm)	PDI
Day 0	33.0	0.284	40.4	0.281	39.0	0.358
Day 4	35.3	0.369	44.1	0.300	49.5	0.363
Day 8	50.4	0.321	37.4	0.372	37.3	0.394
Day 12	37.5	0.298	42.3	0.360	37.5	0.303
Day 16	30.2	0.289	33.8	0.388	36.2	0.316
Day 20	46.0	0.399	45.2	0.315	39.4	0.302

[a] Number average diameter and polydispersity index (PDI) were measured by DLS, showing no statistically significant difference between day 0 with days 4-20.

Supplementary Table 6. Effect of BSA on $PPSU_{20}$ self-assembly[a]

FITC-BSA (mg mL^{-1})	Diameter (nm)	Polydispersity Index	Zeta Potential (mV)
0	61.9	0.24	-53.7±0.5
0.1	60.3	0.17	-51.4±0.7
1.0	3610.3	0.07	-43.0±0.7
10	151.4	0.43	-44.8±1.5

[a] $PPSU_{20}$ vesicular nanogels were formed in the presence of varying concentrations of FITC-BSA. Each nanogel sample was assembled from 5 mg of $PPSU_{20}$.

Supplementary References

1. Huang, J. et al. CHARMM36m: an improved force field for folded and intrinsically disordered proteins. *Nature methods* **14**, 71, 71–73 (2017).
2. MacKerell, A. D. et al. All-Atom Empirical Potential for Molecular Modeling and Dynamics Studies of Proteins. *The Journal of Physical Chemistry B* **102**, 3586–3616 (1998).
3. Miyamoto, S. & Kollman, P. A. Settle: An analytical version of the SHAKE and RATTLE algorithm for rigid water models. *Journal of Computational Chemistry* **13**, 952–962 (1992).
4. Hess, B. et al. GROMACS 4: Algorithms for Highly Efficient, Load-Balanced, and Scalable Molecular Simulation. *Journal of Chemical Theory and Computation* **4**, 435–447 (2008).
5. Essmann, U. et al. A smooth particle mesh Ewald method. *The Journal of Chemical Physics* **103**, 8577–8593 (1995).
6. Hess, B. P-LINCS: A Parallel Linear Constraint Solver for Molecular Simulation. *Journal of Chemical Theory and Computation* **4**, 116–122 (2008).
7. Ortony, J. H. et al. Water Dynamics from the Surface to the Interior of a Supramolecular Nanostructure. *Journal of the American Chemical Society* **139**, 8915–8921 (2017).
8. Qiao, B. et al. Molecular Origins of Mesoscale Ordering in a Metalloamphiphile Phase. *ACS Central Science* **1**, 493–503 (2015).
9. Mark, P. & Nilsson, L. Structure and Dynamics of the TIP3P, SPC, and SPC/E Water Models at 298 K. *The Journal of Physical Chemistry A* **105**, 9954–9960 (2001).
10. Strader, M. L. & Feller, S. E. A Flexible All-Atom Model of Dimethyl Sulfoxide for Molecular Dynamics Simulations. *The Journal of Physical Chemistry A* **106**, 1074–1080 (2002).
11. Qiao, B. et al. Liquid worm-like and proto-micelles: water solubilization in amphiphile–oil solutions. *Physical Chemistry Chemical Physics* **20**, 12908–12915 (2018).

12. Reichardt, C. “Solvents and Solvent Effects in Organic Chemistry” (Wiley-VCH Publishers, 3rd ed., 2003).
