# Supplementary material for: Homopolymer self-assembly of poly(propylene sulfone) hydrogels via dynamic noncovalent sulfone–sulfone bonding
Source: Nat Commun. 2020 Sep 29;11:4896. doi: 10.1038/s41467-020-18657-5 (PMC7525563; doi:10.1038/s41467-020-18657-5)
Supplement: Supplementary file 3 — Description of Additional Supplementary Files [file 41467_2020_18657_MOESM3_ESM.pdf]

Description of Additional Supplementary Files

File Name: Supplementary Movie 1
Description: **Dynamics of one polymer chain in the DMSO all-atom MD simulations.** Only one polymer chain was highlighted, and the polymer chain was coiled initially with the end-to-end distance of around 21 Å based on the sulfur atoms, which increased to around 69 Å at 200 ns.

File Name: Supplementary Movie 2
Description: **Rotation animation of the last simulation snapshot in the DMSO solution.**

File Name: Supplementary Movie 3
Description: **Dynamics of one polymer chain in the water all-atom MD simulations.** Only one polymer chain was highlighted, and the initially extended polymer chain became collapsed within 1 ns, and folded at around 5 ns, aggregated at around 7 ns in the dimeric form.

File Name: Supplementary Movie 4
Description: **Rotation animation of the last simulation snapshot in the water system.**

File Name: Supplementary Movie 5
Description: **Loading of FITC-albumin in PPSU nanogels.** 50 µL of $PPSU_{20}$ solutions (25 mg mL^{-1} in DMSO) were added stepwise (10 µL *per* step or 100 µL *per* step) with 100 µL of aqueous FITC-albumin solutions (1.0 mg mL^{-1}) and then one-time with 400 µL of water. Each step was followed by vortexing to thoroughly mix the samples. FITC-albumin-loaded nanogels were obtained by centrifugation (16,000 g, 10 min).
